# ADAM10 Expression by Ameloblasts Is Essential for Proper Enamel Formation

**DOI:** 10.3390/ijms252313184

**Published:** 2024-12-07

**Authors:** Shifa Shahid, Yuanyuan Hu, Fatma Mohamed, Lara Rizzotto, Michelle C. Layana, Daniel T. Fleming, Petros Papagerakis, Brian L. Foster, James P. Simmer, John D. Bartlett

**Affiliations:** 1 Division of Biosciences, College of Dentistry, Ohio State University, 305 W, 12th Ave., Columbus, OH 43210, USA; shahid.30@osu.edu (S.S.); fmohamed@umich.edu (F.M.); rizzotto.1@osu.edu (L.R.); layana.2@osu.edu (M.C.L.); foster.1004@osu.edu (B.L.F.); 2 Department of Biologic and Materials Sciences, School of Dentistry, University of Michigan 1011 North University, Ann Arbor, MI 48190, USA; yyhu@umich.edu (Y.H.); jsimmer@umich.edu (J.P.S.); 3 Laboratory of Precision Oral Health and Chronobiology, Faculty of Dentistry, Laval University, Dental Medicine Pavilion, 2420, rue de la Terrasse, Quebec City, QC G1V 0A6, Canada; petros.papagerakis@fmd.ulaval.ca

**Keywords:** ameloblast migration, *Cre* Lox recombination, occlusal enamel, cervical enamel, density, hardness, volume, mouse

## Abstract

ADAM10 is a multi-functional proteinase that can cleave approximately 100 different substrates. Previously, it was demonstrated that ADAM10 is expressed by ameloblasts, which are required for enamel formation. The goal of this study was to determine if ADAM10 is necessary for enamel development. Deletion of *Adam10* in mice is embryonically lethal and deletion of *Adam10* from epithelia is perinatally lethal. We therefore deleted *Adam10* from ameloblasts. Ameloblast-specific expression of the Tg(*Amelx*-i*Cre*)872pap construct was confirmed. These mice were crossed with *Adam10* floxed mice to generate *Amelx-*i*Cre*; *Adam10^fl/fl^* mice (*Adam10* cKO). The *Adam10* cKO mice had discolored teeth with softer than normal enamel. Notably, the *Adam10* cKO enamel density and volume were significantly reduced in both incisors and molars. Moreover, the incisor enamel rod pattern became progressively more disorganized, moving from the DEJ to the outer enamel surface, and this disorganized rod structure created gaps and S-shaped rods. ADAM10 cleaves proteins essential for cell signaling and for enamel formation such as RELT and COL17A1. ADAM10 also cleaves cell-cell contacts such as E- and N-cadherins that may support ameloblast movement necessary for normal rod patterns. This study shows, for the first time, that ADAM10 expressed by ameloblasts is essential for proper enamel formation.

## 1. Introduction

Enamel formation is an intricate process that proceeds in three major consecutive developmental stages: presecretory, secretory, and maturation. Ameloblasts are highly specialized cells that dictate the progression of enamel development by altering their function and shape. In the presecretory stage, preameloblasts differentiate and extend cellular projections through the disintegrating basement membrane and into the underlying predentin [1]. The secretory stage starts when amelogenin is first expressed, which occurs near the onset of dentin mineralization. Subsequently, ameloblasts elongate, move away from the dentin as they secrete proteins, and lengthen enamel ribbons that become crystallites. Each ameloblast secretes approximately 10,000 crystallites that will merge into a single enamel rod [2]. The mineralized enamel rod embodies the migratory path of the moving ameloblasts, resulting in a decussating rod pattern in rodent incisors. Once the enamel layer has reached full thickness, mass secretion of the enamel proteins and movement of ameloblasts ceases as secretory ameloblasts transition into the maturation stage, where enamel matrix proteins are removed and enamel hardens into its final form [3,4].

Matrix metalloproteinases are zinc-dependent endopeptidases that cleave extracellular matrix molecules. Matrix metalloproteinase 20 (MMP20; enamelysin) is a tooth specific proteinase essential for healthy enamel development, as mutations in *MMP20* are known to cause enamel malformation [5,6]. Due to its activity on cell-cell junction proteins, MMP20 was suggested to facilitate ameloblast cell migration [7]. However, because of the complexity of ameloblast cell movement, other proteinases are likely to be involved.

A Disintegrin And Metalloproteinases (ADAMs) are a family of membrane-bound zinc-dependent proteinases that are classified as sheddases because they cleave/shed extracellular domains of membrane bound proteins [8,9]. Previously, a positive expression screen of six proteolytically active ADAM family members was reported in the developing mouse enamel organ. Among the six ADAMs, expression of *Adam10* was observed in the apical loop containing the stem cells that become pre-ameloblasts and then ameloblasts as they migrate with the continuously erupting incisor. *Adam10* expression persists throughout the secretory stage in rodent incisors. Strikingly, expression of *Adam10* predominantly ceases at the end of secretory stage when ameloblasts movement also ends [10]. Notably, ADAM10 sheds/cleaves about 100 substrates [11], including cell-cell adhesion proteins [12,13], and by cleaving these adhesion proteins, ADAM10 can facilitate cell migration/invasion [8,9], which makes it a candidate for facilitating ameloblast movement necessary to form the decussating enamel rod pattern. Additionally, ADAM10 sheds cell-signaling proteins from cell surfaces. The best-characterized ADAM10 cell signaling substrate is Notch. However, Notch-1, -2, and -3 proteins are not expressed on pre-ameloblasts or ameloblasts [14]. But, among the almost 100 substrates ADAM10 sheds [11] and that both pre-ameloblasts and ameloblasts express, are the hyaluronic acid receptor CD44 [15], EGFR [16], ERBB2 [17], RELT [18], and COL17A1 [19,20]. Therefore, ADAM10 is also a candidate for regulating cell signaling during enamel development.

A previous study [21] used Keratin14*^Cre/+^*; *Adam10^fl/fl^* mice to demonstrate *Notch 1* downregulation in dental epithelium with subsequent loss of epithelial cell boundaries. This Keratin14 promoter strategy to drive the *Cre* recombinase had the advantage of knocking out *Adam10* expression from its onset in the apical loop of incisors. However, this experimental approach confounded interpretation of the results since over 95% of the Keratin14*^Cre/+^*; *Adam10^fl/fl^* mice die within the first 24 h of life, likely due to perturbed skin barrier function causing transdermal water loss [22] (p. 497). Since visible enamel defects result from as little stress as occurs with a fever [23,24], and especially since systemic illnesses are known to interfere with normal enamel deposition [25], we generated an *Adam10* conditional knockout mouse using an amelogenin promoter-driven i*Cre* recombinase [26] (*Amelx-*i*Cre*; *Adam10^fl/fl^*). This mouse knocks out ADAM10 expression in ameloblasts starting at the onset of the secretory stage of amelogenesis and therefore avoids the stress of lethal transdermal water loss associated with the Keratin14*^Cre^*^/+^; *Adam10^fl/fl^* mice. Provided here are detailed analyses of enamel formation when *Adam10* is ablated specifically from ameloblasts in healthy mice that do not suffer systemic illness and perinatal death.

## 2. Results

### 2.1. Amelx-iCre Mice Start Expressing Cre Recombinase in Secretory Ameloblasts

**Figure 1:**
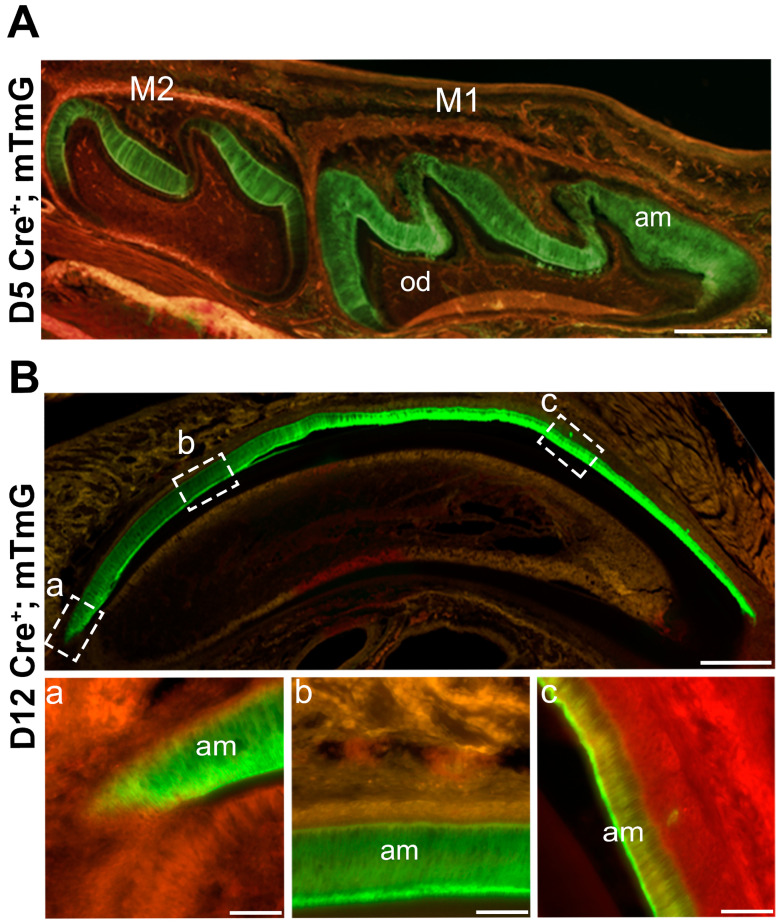
*Amelx-*i*Cre* mice express *Cre* recombinase only in ameloblasts. *Amelx-*i*Cre* mice were crossed with the mouse mT/mG reporter strain so that *Cre* expression would cause tissues to stain with green fluorescent protein (GFP). (**A**) GFP staining was restricted to ameloblasts in molars at postnatal day 5 whereas areas of red florescence indicate no *Cre* recombination. Scale bar 200 µm. (**B**) In postnatal day 12 maxillary incisor sections, amelogenin promoter driven i*Cre* expression starts at the onset of the secretory stage (**a**) and persists throughout the length of the incisor where ameloblasts are present (**b**,**c**). Scale bar 1000 µm. M1, first molar; M2, second molar; am, ameloblasts.

Previously, five different *Amelx promoter driven Cre* mouse lines were developed that expressed improved *Cre* (i*Cre*) recombinase within a large (250 kb) bacterial artificial chromosome DNA vector [26]. After preliminary testing, the Tg(*Amelx*-i*Cre*)872pap mice were chosen for further analyses by assessing the tissue specific expression of this i*Cre* construct. The *Amelx-*i*Cre* mice were bred with ROSA^mT/mG^ mice to generate *Amelx*-i*Cre*; mT/mG mice. Without *Cre* recombination, these mice express tdTomato (mT), which generates red fluorescence in tissues (Figures S1 and S2). When the *Cre* recombinase is expressed, mT is spliced out and tissues are stained with green fluorescent protein (mG). Fluorescent imaging of post-natal day 5 molar cryosections revealed strong mG staining in ameloblasts from the first and second mandibular molars (Figure 1A). In contrast, expression of mT was detected in odontoblasts, alveolar bone, and the remaining layers (stratum intermedium, stellate reticulum, outer epithelium) of the enamel organ, indicating that *Cre*-mediated recombination did not occur in these tissues. Murine incisor cryosections at post-natal day 12 yielded similar results. Positive mG expression was observed in secretory stage ameloblasts, which persisted in maturation-stage ameloblasts (Figure 1B).

### 2.2. Assessment of Amelx-iCre Expression in Multiple Tissues and Quantification of Adam10 Expression in the Amelx-iCre; Adam10^fl/fl^ (Adam10 cKO) Mouse Incisors

**Figure 2:**
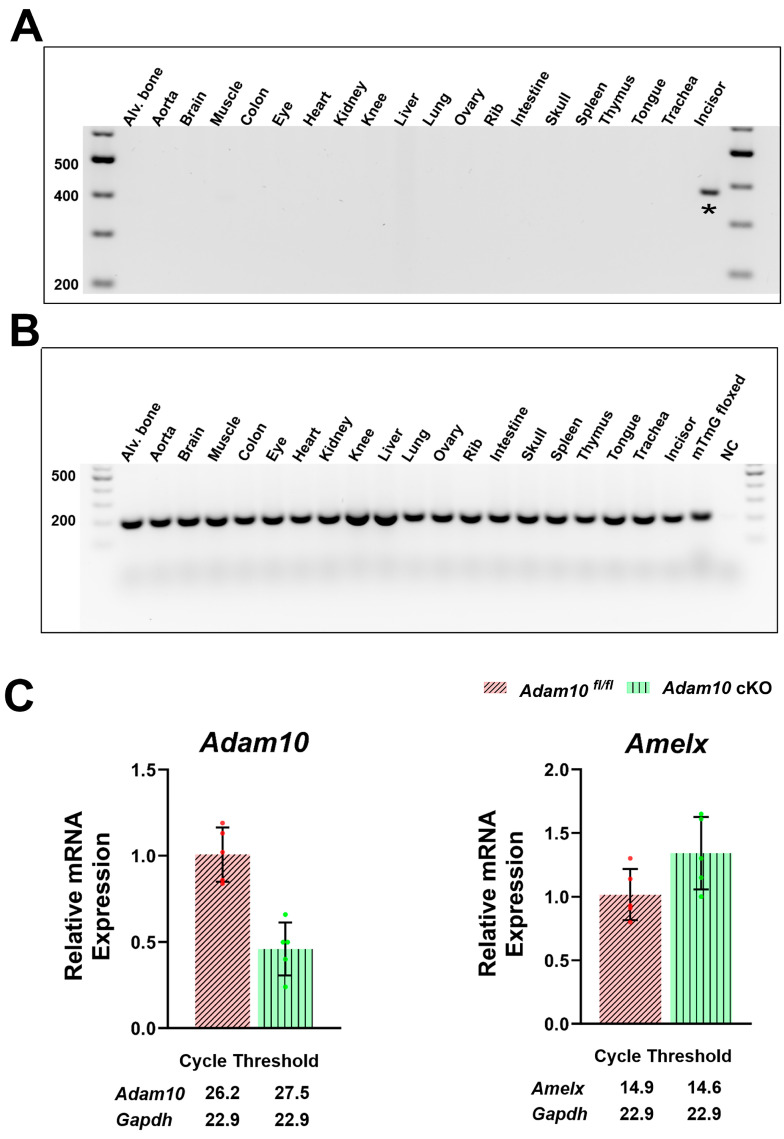
Genomic PCR analysis to detect the presence or absence of *Amelx-*i*Cre* mediated recombination in multiple mouse tissues and quantification of *Adam10* and amelogenin expression. (**A**) A 376 base pair mG product results from the *Cre*-recombined construct, while a (**B**) 200 base pair product results from the mT region that was not recombined by *Amelx*-i*Cre.* Of the 20 tissues assessed, only the whole incisor demonstrated *Amelx-*i*Cre* mediated recombination. (**C**) Quantification of *Adam10* and amelogenin (*AmelX*) expression by qPCR analyses of 5-day old first molar enamel organs. The *Adam10* cKO was expressed at significantly lower levels than was the *Adam10^fl/fl^* control, demonstrating that the preponderance of the *Adam10* genes in the ameloblast layer were successfully excised. In contrast, no significant difference was observed in native amelogenin gene expression between the *Adam10* cKO and *Adam10^fl/fl^* control mice, indicating that the *Amelx-*i*Cre* construct did not affect native amelogenin expression. Dots along the error bars represent the results of individual samples. Dots along the error bars represent the results of individual samples.

To confirm ameloblast-specific i*Cre* expression, genomic PCR analyses in multiple tissues were performed using primers that can amplify a 376-base pair mG product only if i*Cre* recombination has occurred [27]. A total of 20 tissues were tested (alveolar bone, aorta, brain, muscle, colon, eye, heart, kidney, knee, liver, lung, ovary, rib, intestine, skull, spleen, thymus, tongue, trachea, and whole incisor). *Cre* activity was detected only in the incisor (Figure 2A), demonstrating that *Amelx-*i*Cre* activation is tooth-specific. To further confirm tooth-specific i*Cre* expression, genomic PCR analyses were performed in the same tissues using primers that can amplify a 200-base pair mT product only if i*Cre*-recombination has not occurred [27]. The presence of the 200 base pair mT product revealed that in the tissues examined, including whole incisors containing bone dentin and enamel, i*Cre* recombination did not occur (Figure 2B). Taken together with the results of Figure 1, whole incisor ameloblasts displayed *Cre* recombination, but the associated incisor tissues (bone and dentin) did not. These data confirm that i*Cre* recombination occurs only in the ameloblasts of the mouse enamel organ and not in the other 19 tissues examined.

The *Amelx*-i*Cre*; mT/mG mice confirmed that ameloblasts express the *Amelx*-i*Cre* transgene. The *Amelx*-i*Cre* mice were therefore crossed with *Adam10^fl/fl^* mice to generate *Amelx*-i*Cre*; *Adam10^fl/fl^* mice (*Adam10* cKO). Quantitative real-time PCR (qPCR) analyses of 5-day old first molars that were predominantly in the secretory stage of enamel development revealed that *Adam10* expression was significantly downregulated in the *Adam10* cKO enamel organs, indicating that the preponderance of *Adam10* genes in the ameloblast layer were successfully excised (Figure 2C). Note that for this analysis, the stratum intermedium, stellate reticulum, and outer enamel epithelium were present along with the cKO ameloblasts and that the pulp organ was not completely removed from the assessed day-5 enamel organs, which means these tissues contributed to the *Adam10* cKO expression level shown in Figure 2C.

To assess whether the *Amelx*-i*Cre* construct affected expression of the endogenous amelogenin gene in *Adam10* cKO mice, qPCR of amelogenin gene expression in the cKO mice and controls was performed. No significant difference was observed in endogenous amelogenin gene expression between the two genotypes assessed (Figure 2C). Therefore, any observed phenotype discovered in the cKO mice would be attributed to the loss of *Adam10* expression by secretory ameloblasts.

### 2.3. Phenotypic Assessment of Teeth by Genotype

**Figure 3:**
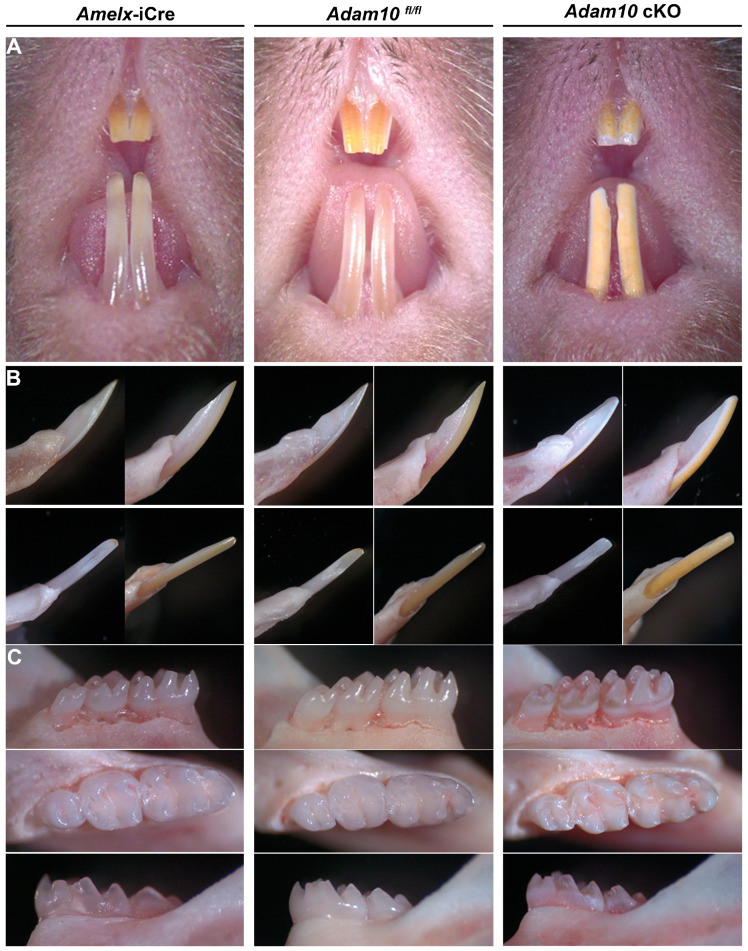
Phenotypic assessment of *Amelx*-i*Cre*, *Adam10^fl/fl^*, and *Adam10* cKO incisors plus molars from mice at 7 weeks of age. (**A**) Frontal view of maxillary and mandibular incisors. The *Adam10* cKO incisor enamel appears to be dull, rough, pigmented, and abraded. (**B**) Mesial (**upper left**), lateral (**upper right**), lingual (**lower right**), and labial (**lower left**) views of mandibular incisors. The incisal edge of *Adam10* cKO incisors were blunted due to attrition. (**C**) Buccal, occlusal, and lingual views of mandibular molars. *Adam10* cKO molars appear rough, discolored, and show distinct chalky banding near the cervical edge. These molar cusps are rounded and show signs of attrition. Teeth from *Amelx*-i*Cre* and from *Adam10*^fl/fl^ mice appear normal in shape and color.

*Adam10* cKO incisors at 7 weeks show abrasion on the labial surface of the maxillary incisors (Figure 3A). The mandibular incisors were blunted, displayed a yellow discoloration, and had a rough sandpaper-like surface when compared to the smooth translucent enamel observed in both *Amelx-*i*Cre* and *Adam10^fl/fl^* mice (Figure 3B)*. Adam10* cKO molars also presented with a rough enamel texture with signs of occlusal wear on the cusps. A distinct chalky white band was present near the cervical margin of the cKO molars. In contrast, the *Amelx-*i*Cre* and *Adam10^fl/fl^* molars appeared normal (Figure 3C).

### 2.4. Quantification of Incisor Enamel Hardness

**Figure 4:**
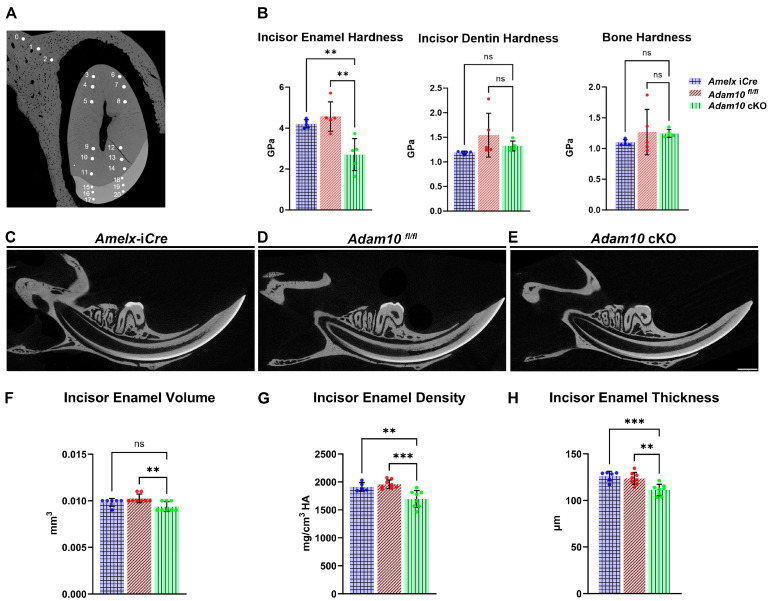
Quantification of incisor enamel hardness, volume, density and thickness. (**A**) Shown are nanohardness indents of enamel, dentin and alveolar bone that were performed on *Amelx*-i*Cre*, *Adam10^fl/fl^*, and *Adam10* cKO mice. Each sample is indented at 3 positions in bone (positions 0–2), 12 positions in dentin (3–14) and 6 positions in enamel (15–20). (**B**) Bar graph of the mean nanohardness (GPa) values show the *Adam10* cKO mice had significantly reduced enamel hardness compared to controls. In contrast, dentin and bone nanohardness levels were not significantly different among the genotypes tested. (**C**–**E**) 2D renderings from µCT analyses of adult hemi-mandibles in sagittal view. The mineralized enamel appeared less dense in *Adam10* cKO incisors compared to controls. (**F**–**H**) Compared to *Amelx*-iCre and *Adam10^fl/fl^* controls, *Adam10* cKO mouse incisors exhibit 10% reduced enamel mineral density and 10% reduced enamel thickness Dots along the error bars represent the results of individual samples (*** *p* < 0.001, ** *p* < 0.01, ns; non-significant).

Nanoindentation was performed on mandibular hemi-mandibles sectioned at the buccal alveolar crest (Figure 4A). These analyses showed that, compared to *Amelx-*i*Cre* and *Adam10^fl/fl^* incisors, *Adam10* cKO incisors were significantly softer (** *p* < 0.01) than normal (Figure 4B). The average hardness value of the cKO incisors was 2.7 GPa, which reflects a 40% decrease compared to hardness values of *Amelx-*i*Cre* and *Adam10^fl/fl^* incisors (4.2 and 4.6 GPa, respectively). Among the three genotypes of mandibular incisors, no statistically significant nanohardness differences were observed in dentin or alveolar bone (Figure 4B), demonstrating that only the enamel was affected in the *Adam10* cKO mice.

### 2.5. Micro Computed Tomography (µCT) Analyses

Next, µCT was performed to determine if volumetric and mineralization defects were present in the cKO incisor enamel. High-resolution sagittal images (Figure 4C–E) of mandibular incisors revealed a small decrease in incisor enamel volume with a 10% decrease in mineral density plus a 10% reduced enamel thickness in cKO incisors at the buccal alveolar crest compared to *Amelx-*i*Cre* and *Adam10^fl/fl^* controls (Figure 4F–H).

**Figure 5:**
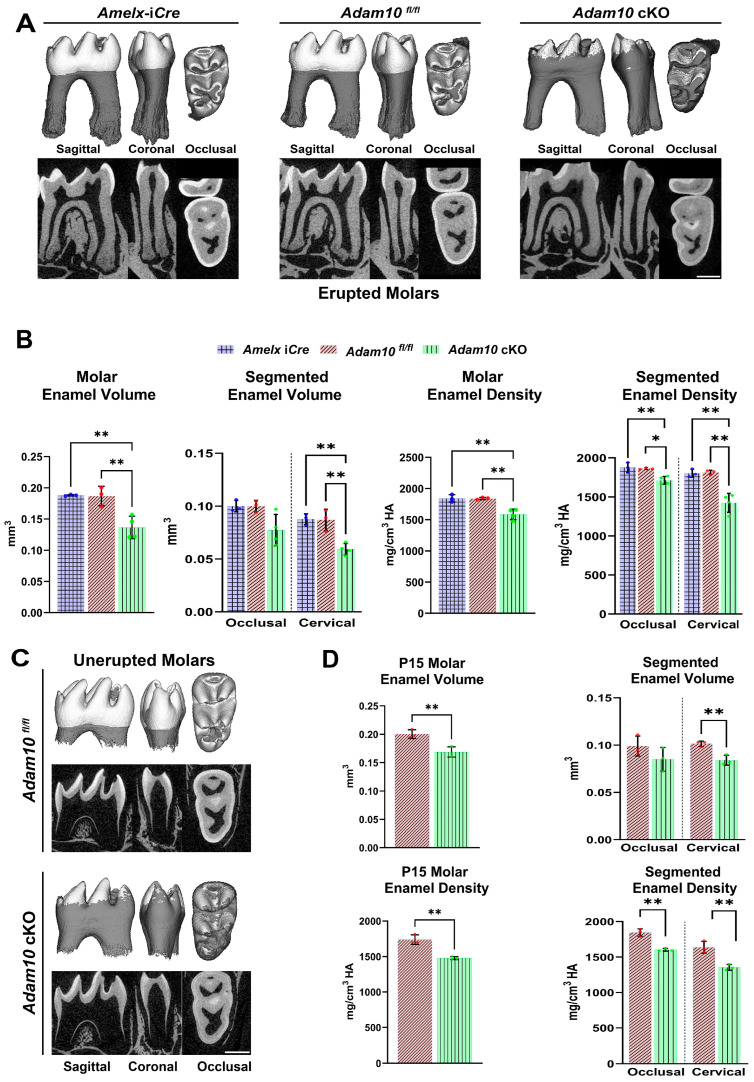
2D and 3D renderings from µCT analyses of erupted and unerupted mandibular first molars in *Adam10* cKO mice and controls. (**A**) Compared to controls, deletion of *Adam10* leads to a poorly defined and under mineralized enamel layer. Sagittal and coronal views reveal flattening of *Adam10* cKO cusps suggesting that these molars have undergone attrition. Occlusal views of *Adam10* cKO molars show a severely hypomineralized enamel layer with reduced density as observed by the worn (3D) and darker (2D) enamel. (**B**) Quantitative analyses of total molar enamel volume and density show approximately a 27% decrease in volume and a 14% reduction in mineral density compared to controls. Segmentation of molars into occlusal and cervical regions reveal a greater reduction of volume and density at the cervical region of *Adam10* cKO molars when compared to the *Amelx*-i*Cre*, and *Adam10^fl/fl^* molars. (**C**) 2D and 3D renderings from µCT analyses of unerupted mandibular molars at postnatal day 15 (P15). Like erupted molars, *Adam10* cKO unerupted molars exhibit significantly reduced enamel volume and density. Enamel in the cervical region is present but is severely undermineralized. (**D**) For the unerupted molars, quantitative measurements confirmed an overall 16% and 15% reduction of total enamel volume and density respectively. A greater decrease in cervical enamel volume versus occlusal volume (17% versus 14%) and cervical density versus occlusal density (17% versus 13%) was also confirmed in *Adam10* cKO unerupted molars. Note that the total decrease in enamel volume for the *Adam10* cKO erupted molars was 27% while that for the unerupted molars was 16%. This difference may represent the level of attrition in the 7-week-old erupted molars. Dots along the error bars represent the results of individual samples (** *p* < 0.01, * p < 0.05).

Enamel density and volume in *Adam10* cKO mandibular first molars were also compared to molars from the control *Amelx-*i*Cre* and *Adam10^fl/fl^* mice. At 7-weeks of age, signs of occlusal wear were observed on cusps tips from cKO first molars, indicating loss of enamel through abrasion. The enamel layer was hypomineralized and could not be distinguished from dentin by µCT settings at the 1600 mg HA/cm^3^ threshold (Figure 5A). Using a combination of semi-automatic and manual corrections, the *Adam10* cKO enamel layer was traced, which identified a 27% reduction in enamel volume and a 14% decrease in mineral density (Figure 5B).

Molar crowns were then subdivided into cervical and occlusal sections to quantify enamel volume and density at these locations. Occlusal enamel volume was significantly reduced by 23% in the cKO molars compared to controls, and the cervical enamel volume in cKO molars had a greater reduction of 32% relative to controls. Assessment of segmented mineral density revealed that the cKO mice had an 8% reduction in occlusal mineral density and that the cKO cervical enamel had a 21% reduced mineral density compared to the corresponding densities of control *Amelx-*i*Cre* and *Adam10^fl/fl^* molars (Figure 5B). This demonstrates that the chalky white banding pattern observed in the cervical region of cKO molars (Figure 3C) has both a decreased volume and density relative to the occlusal region of the same cKO molars.

Next, volume and density were quantified in mandibular first molars at post-natal day 15 (P15). At P15, enamel formation on the first molars is virtually complete but the molars have not fully erupted into the oral cavity. Unlike erupted molars, no signs of attrition were observed in the unerupted P15 molars. Like erupted molars, unerupted cKO molars exhibited a significant reduction of total enamel volume (16%) and density (15%) versus controls (Figure 5C,D). Also, as with the erupted molars, the differences versus controls were reduced more in the cervical area of the unerupted molars (volume 17% and density 17%) than the occlusal regions (volume 14% and density 13%) compared to the corresponding control molar locations (Figure 5D).

### 2.6. Backscatter Scanning Electron Microscopy (bSEM) Assessment of Ultrastructural Changes in Adam10 cKO Incisors

**Figure 6:**
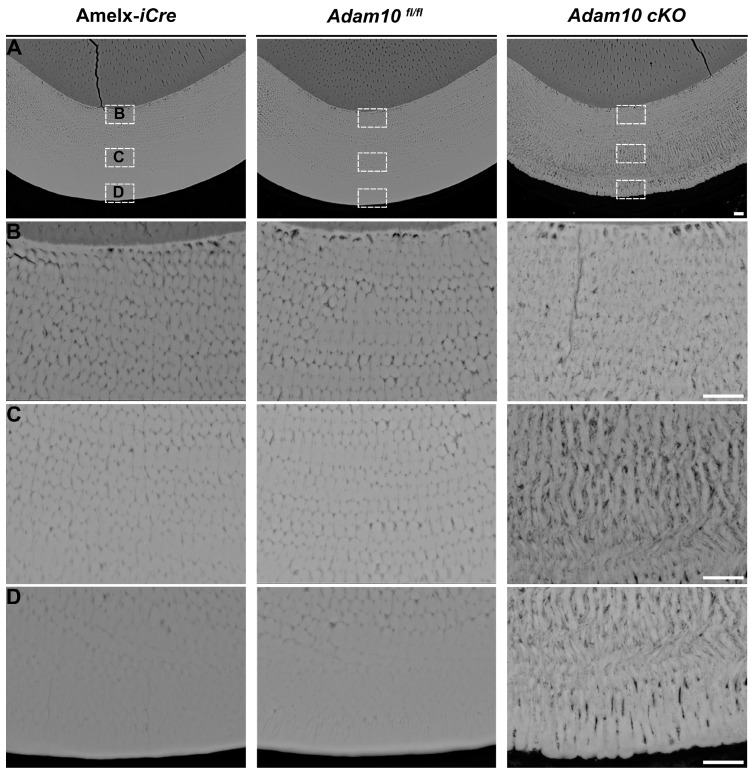
Backscatter SEM of 7-week mandibular incisor cross sections from *Amelx*-i*Cre*, *Adam10^fl/fl^*, and *Adam10* cKO mice. (**A**) Panels show cross-sections of incisor enamel at the buccal alveolar crest where the enamel layer is near to erupting into the oral cavity. Normal enamel thickness is present in *Amelx*-i*Cre*, and *Adam10^fl/fl^* incisors, but the *Adam10* cKO enamel is darker indicative of increased protein content and appears thinner than normal. Scale bar 10 µm. (**B**–**D**) Higher magnification (2000×) reveals distinct enamel rod decussation patterns. Compared to the *Amelx*-i*Cre* and *Adam10^fl/fl^* enamel, the rod/interrod pattern of *Adam10* cKO enamel is somewhat disorganized near the dentin-enamel junction, but the enamel rods become progressively more S-shaped and loosely packed (porous) toward the outer enamel edge. Scale bar 10 µm.

Incisor enamel at the level of the buccal alveolar crest was examined by bSEM. The enamel layers from *Amelx-*i*Cre* and *Adam10^fl/fl^* control incisors were highly mineralized and of normal thickness. In contrast, the enamel from *Adam10* cKO incisors was less thick and appeared porous (Figure 6A). Distinctively organized and decussating enamel rods were observed in *Amelx-*i*Cre* and *Adam10^fl/fl^* incisors, whereas the rod-interrod pattern in cKO incisors was loosely arranged and disorganized with apparent S-shaped rods and spaces between these rods (Figure 6C,D). The rod pattern disorganization became progressively more severe in cKO incisors from the dentin-enamel junction (DEJ) to the incisal edge (Figure 6B–D).

### 2.7. Histological Assessment of Stratum Intermedium and Ameloblasts in Adam10 cKO Incisors

**Figure 7:**
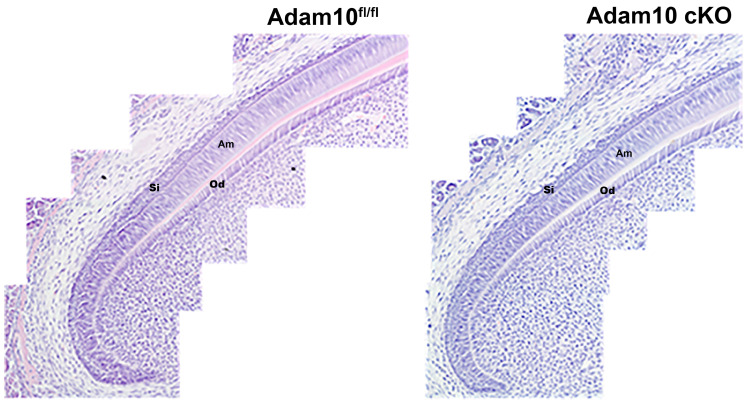
Histological comparison of incisor ameloblasts (Am) and stratum intermedium (Si) from *Adam10^fl/fl^* (control) and *Adam10* cKO (experimental) mice. Compared to the *Adam10^fl/fl^* incisor ameloblasts, the *Adam10* cKO ameloblasts appeared to be normally aligned and did not significantly differ from those present in the *Adam10^fl/fl^* mice. Perhaps this is not unexpected since some level of ameloblast alignment would likely be necessary to generate a rod pattern even if that pattern was disorganized. Both the ameloblast and stratum intermedium tissue layers appeared normal. Od, odontoblast layer.

Incisors from 7-week-old *Adam10^fl/fl^* and *Adam10* cKO mice were sectioned and H&E stained. Figure 7 shows representative samples from control and *Adam10* cKO mice. In contrast to a previous study [21], no obvious differences were observed between genotypes in the structure of the stratum intermedium (Si) or the ameloblast (Am) tissue layers. Although the enamel rod pattern in the *Adam10* cKO *Incisors* was disrupted, a rod pattern was nevertheless present, and this would require a certain level of ameloblast organization to form the disrupted enamel rod pattern.

### 2.8. Quantification of Col17a1, Relt, Ambn Enam and Mmp20 Gene Expression Levels by Genotype

To determine if ADAM10-mediated cell signaling effects the expression of genes that, when mutated, can cause enamel defects, a preliminary study was performed by use of qPCR to quantify the expression levels of secretory stage enamel matrix genes (*Ambn, Enam*, *Mmp20*) and quantify expression levels of genes encoding cell surface proteins (*Col17a1*, *Relt*) that ADAM10 is known to cleave. Day-5 first molar mRNA from *Adam10^fl/fl^* controls was extracted to compare gene expression levels with mRNA from *Adam10* cKO mouse first molars. Compared to the *Adam10^fl/fl^* control, no significant difference in expression was observed among the genes encoding enamel matrix or cell surface proteins when compared to the expression levels observed in the *Adam10* cKO mice (Figure S3). Further investigations are necessary to determine if ADAM10-mediated cell signaling plays a role in the enamel phenotype observed in *ADAM10* cKO mice.

## 3. Discussion

ADAM10 is a multifunctional protease that attaches to the cell surface via a transmembrane domain. Its catalytic domain is positioned extracellularly so that it can shed proteins from the cell surface [28,29]. RELT is a receptor of the tumor necrosis factor super family that, when mutated, can cause amelogenesis imperfecta [18]. Previously, we demonstrated in vitro that ADAM10 cleaves the RELT extracellular domain and we suggested that absence of ADAM10 may cause an abundance of RELT to remain on the ameloblast cell surface, which could result in enamel malformation [10]. However, an engineered *Relt* mutation in mice caused a milder enamel phenotype than was observed here in *Adam10* cKO mice [18]. ADAM10 also cleaves COL17A1, which likewise is a transmembrane protein that when mutated causes enamel defects in human teeth [20]. Like *Adam10* cKO mice, *Col17a1^−/−^* mice have an irregular enamel rod pattern and abraded teeth indicating defective mineralization [19]. However, it is difficult to make conclusions about a protease’s contribution to enamel formation by examining the functional loss of a potential substrate. If ADAM10 cleaves the substrate, the absence of ADAM10 allows the substrate to remain attached to the cell surface and this phenotype may be different from what occurs when the substrate is nonfunctional.

ADAM10 is transported to the cell surface by a class of tetraspanins (Tspans) termed TspanC8s because they have 8 cysteines in their extracellular domain. Six different TspanC8s are capable of transporting ADAM10 to the cell surface. Once there, the TspanC8 remains complexed with ADAM10 at the cell surface. So, each TspanC8 selects the ADAM10 location and thereby modulates ADAM10 substrate selectivity [30,31]. Additionally, each TspanC8 may influence ADAM10 substrate specificity, perhaps through conformational interactions, which likely contributes to the ability of ADAM10 to cleave approximately 100 substrates [11]. Previously, mouse enamel organs were screened for the presence of ADAM proteinases. *Adam10* was the only ADAM expressed during the secretory stage, when ameloblasts move relative to each other, but not during the maturation stage, when ameloblasts stop moving. Enamel rods are the mineralized path of ameloblast cell movement and since ameloblasts lacking ADAM10 have malformed S-shaped rod patterns, this suggests that ADAM10 plays a role in ameloblast movement during the secretory stage of enamel development. Therefore, it is possible that a TspanC8 transports ADAM10 to the ends of each ameloblast cohort to detach them from adjacent cohorts. This may facilitate groups of ameloblasts to slide by one another, which is necessary to form the decussating enamel rod pattern characteristic of rodent incisor enamel [32,33]. Here, ameloblast incisor and molar histology sections were examined and the cKO ameloblasts were apparently normally aligned (Figure 7). However, at least some level of ameloblast alignment would likely be necessary to generate a rod pattern even if that pattern was disorganized compared the wild-type rod pattern. It is also possible that the cKO mineralizing front is disrupted such that the ameloblast Tomes processes do not produce enamel ribbons in an organized manner.

Since ADAM10 plays a significant role in regulating cell signaling [9,34,35], another possibility is that lack of ADAM10 resulted in disrupted cell signaling within the enamel organ, which caused the dysplastic phenotype observed in the cKO mice. The finding that the cervical region of the cKO molars had less volume and reduced density compared to the occlusal region was unexpected. However, since enamel development begins at the cusp tip and progresses to the cervical regions, this is consistent with the latest formed enamel becoming more adversely affected by a lack of ADAM10 than the earliest formed enamel. Note that the latest formed outer layers of incisor enamel were more disrupted than the inner layers (Figure 6). This suggests that ADAM10 function becomes increasingly more important as enamel development progresses and lends support to the possibility that cell signaling is disrupted in the enamel organ. This is because the loss of appropriate cell signaling could become cumulative as enamel development progresses, resulting in a more dysplastic phenotype near the end of enamel development as compared to the beginning. However, our preliminary investigations into gene regulation of enamel matrix proteins and ADAM10 cell surface substrates (Figure S3) revealed no significant differences in their transcript levels between control and cKO mouse enamel organs. Further studies are necessary to identify the mechanistic function of ADAM10 during enamel development.

In addition to perinatal lethality [22], another difference between this and the previous *Adam10* conditional knockout study [21] is the timing of when *Adam10* was knocked out. Keratin14 is expressed in the first branchial arch of the oral and tooth ectoderm [36]. Therefore, Keratin14^Cre/+;^ *Adam10^fl/fl^* mice delete *Adam10* at the earliest stages of tooth development in all epithelial tissues. However, this study shows that when *Adam10* is deleted specifically from secretory stage ameloblasts, the resulting enamel is malformed. Notably, in contrast to our findings, the Keratin14^Cre/+;^ *Adam10^fl/fl^* study [21] also examined *Amelx*^Cre/+^; *Adam10^fl/fl^* mice and described the enamel as forming normally. However, the authors did not identify which of the five available *Amelx*^Cre/+^ constructs they used, and a figure in their supplementary data showing *Amelx*^Cre/+^; R26^mTmG^ results indicated that very few ameloblasts would have deleted *Adam10* from their nuclei because only a few were colored green due to excision of the TdTomato region by the *Cre* recombinase.

Therefore, in addition to the proteinases MMP20 (matrix metalloproteinase-20; enamelysin) and KLK4 (Kallikrein-4; enamel matrix serine protease-1), which are essential for enamel formation in both humans and mice [3], this study demonstrates that the proteinase ADAM10 also plays a role in enamel formation in mice. A limitation of the study is that it may never be known if ADAM10 is necessary for human enamel development because its homozygous inactivation is embryonically lethal.

## 4. Conclusions

The Tg(*Amelx*-i*Cre*)872pap construct was expressed only in secretory ameloblasts, demonstrating its suitability to eliminate *Adam10* expression in these cells. *Adam10* cKO mice have teeth that are discolored and abraded, and their enamel is softer than normal. Notably, the cKO enamel density and enamel volume were significantly reduced in both incisors and molars. Moreover, the enamel rod pattern became progressively more S-shaped and disorganized moving from the DEJ to the outer enamel surface and this disorganized rod structure created gaps between and among the rods, suggesting that the ameloblasts do not move properly, or perhaps signal properly, to form a normal rod pattern. Future studies are necessary to interrogate the mechanisms of how ADAM10 functions to facilitate healthy dental enamel formation. Perhaps by examining ADAM10 shedding of critical receptors such as RELT or cell-cell contact proteins such as COL17A1 and E-, N-cadherins, additional mechanistic clues will be discovered.

## 5. Materials and Methods

### 5.1. Mice

All animals used in this study were housed in Association for Assessment and Accreditation of Laboratory Animal Care International (AAALAC) accredited facilities and were treated humanely based on protocols approved by the Ohio State University Institutional Animal Care and Use Committee. Approximately equal numbers of male and female adults and pups were examined during the experimental protocols and five or fewer adult mice were kept in each standard cage. The *Amelx-iCre* mice were generated as described previously [26]. These mice were backcrossed into C57BL/6 (wild-type, WT) mice for at least eight generations to obtain a homogeneous genetic background. To confirm *Cre* recombinase activity, *Amelx-*i*Cre* mice were crossed with B6.129(Cg)-*Gt(ROSA)26Sor^tm4(ACTB-tdTomato,-EGFP)Luo^*/J (ROSA*^mTmG^*) mice obtained from Jackson Laboratory (JAX:007676) [37]. To study the effects of *Adam10* deletion in ameloblasts, *Amelx-iCre* mice were mated with B6;129S6-*Adam10^tm1Zhu^*/J (*Adam^fl/fl^*) obtained from Jackson Laboratory (JAX:009357) [38] to generate *Amelx-iCre; Adam10^fl/fl^* (*Adam10* cKO) mice. We used the minimum number of animals necessary to attain statistical significance. Left hemi-mandibles were used for µCT analyses and right hemi-mandibles were used for SEM and Nano-indentation analyses. The number of animals used for each experiment was based on the numbers of animals we have used previously to attain statistical significance for similar experiments [1,10,39].

### 5.2. Detection of Cre-Recombination by Genomic PCR and Assessment of Gene Expression Levels by qPCR

Total DNA was extracted from *Amelx*-i*Cre;* mT/mG mouse tissues using DNeasy Blood & Tissue Kit (Qiagen, Germantown, MD, USA). PCR primer pairs (mT-F: GCAACGTGCTGGTTATTGTG; mT-R: TGATGACCTCCTCTCCCTTG) and (mG-F: GTTCGGCTTCTGGCGTGT; mG-R: TGCTCACGGATCCTACCTTC) amplified non-recombined and *Cre*-recombined alleles generating 200bp and 376bp DNA products, respectively [27].

For quantitative polymerase chain reaction (qPCR), molar enamel organs were extracted from 5-day *Adam10^fl/fl^* and *Adam10* cKO mice. Enamel organs were immediately processed for RNA extraction. Total RNA was isolated using the miRNeasy Micro Kit (Qiagen, Cat. No. 217084). Enamel organs from approximately 1–2 pups were pooled to ensure sufficient concentration of RNA. Following extractions, RNA yield and quality were determined by NanoDrop ND-1000 spectrophotometer. One hundred nanograms of RNA was transcribed into cDNA using the High Capacity cDNA Reverse Transcription Kit (Applied Biosystems, Carlsbad, CA, USA, Cat. No.4368813). Real-time PCR amplification was performed using the TaqMan Fast Advanced Master Mix with optimized probes (Table S1) from Applied Biosystems. Relative expression levels of assessed genes were calculated by 2−ΔΔCt method using *Gapdh* as the reference control gene. Five samples per group were analyzed in triplicate.

### 5.3. Tissue Preparation, Histology and Fluorescence Microscopy

For frozen sections, mouse incisors were harvested from post-natal day 12 mice and first molars were harvested from post-natal day 5 *Amelx*-i*Cre;* mT/mG pups. All other tissues were collected from 7-week-old *Amelx*-i*Cre;* mT/mG mice (n = 3/group). Tissues were fixed in 4% paraformaldehyde at room temperature overnight. Following fixation, maxillae and mandibles were decalcified in 10% EDTA for 5–12 days, embedded in optimal cutting temperature, snap frozen, and sectioned at 10 µm with a cryostat. Soft tissue samples were embedded and sectioned directly after fixation. Sections were rinsed in 1x PBS and stained with DAPI (Life Technologies, Cat No. D1306, 1:5000). Sections were imaged with Lionheart LX microscope (Agilent Technologies, Santa Clara, CA, USA) and Zeiss AXIO Imager.Z2 (Zeiss, White Plains, NY, USA).

Hemimaxillae from post-natal day 12 *Adam10* cKO and *Adam10^fl/fl^* mice were fixed in Bouin’s solution for 24 h, decalcified in 10% EDTA, embedded in paraffin and sectioned at 5 µm. Deparaffinized sections were stained with hematoxylin and eosin (H&E) (n = 3/group).

### 5.4. Phenotypic Assessment of Incisors and Molars

Mandibles (n = 3/group) from 7-week-old mice were stripped of soft tissues and subsequently immersed in 10% protease solution (Sigma-Aldrich, St. Louis, MO, USA) diluted in 1x phosphate-buffered saline (PBS) at 56 °C for 10 min. Teeth were rinsed with 1x PBS, air dried, and photographed using a Leica S9i stereomicroscope (Leica Microsystems, Deerfield, IL, USA).

### 5.5. Nanohardness Testing

Hemi-mandibles (n = 5/group) from *Amelx*-i*Cre*, *Adam10^fl/fl^*, and *Amelx*-i*Cre; Adam10^fl/fl^* mice were collected at 7 weeks. After removing soft tissues, the right-side mandibles were fixed in 4% PFA overnight and dehydrated with acetone gradient (30, 50, 70, 80, 90, and 100%) for 30 min each and embedded in resin (Embed 812, Electron Microscopy Services, Hatfield, PA, USA). The samples were cured at 60 °C for 48 h. Sample discs were cut transversely at the level of the labial crest of the alveolar bone. Samples were re-embedded in Castolite AC in 25-mm SeriForm molds (Struers, Cleveland, OH, USA). Incisor cross sections were polished with waterproof carbide papers and 1-micron diamond paste. Samples were then nanoindented using a Hysitron 950 Triboindenter (Bruker, Allentown, PA, USA) with a nanoDMA transducer and Berkovich probe. Indentations were analyzed using Triboscan 9 software.

### 5.6. Micro-Computed Tomography (µCT)

Left mandibles from adult 7-week-old and post-natal day 15 mice were fixed in 10% neutral-buffered formalin and scanned in a μCT 50 (Scanco Medical AG, CH-8306 Brüttisellen, Switzerland) at 70 kVp, 85 μA, 0.5 mm Al filter, 900 ms integration time, and 6 μm voxel size. Following reconstruction, DICOM files were calibrated to five known densities of hydroxyapatite (mg HA/cm^3^) and analyzed with AnalyzeDirect 14 software. Incisor enamel was segmented at >1600 mg HA/cm^3^ for all groups. Molar enamel was manually traced for *Amelx*-i*Cre; Adam10^fl/fl^* mice [40]. Incisor analyses were performed on 6–8 mice/group and analyses of erupted and unerupted molars were performed on 3–4 mice/group. Samples were randomly numbered and, to enhance validity and reproducibility, the individual performing the scans was blinded as to which mouse genotype was being scanned.

### 5.7. Backscattered Scanning Electron Microscopy (bSEM)

Sample preparation for bSEM was identical to hardness testing (n = 5/group). Incisor cross sections from 7-week old mice were sputter coated with carbon and imaged using JEOL-JSM-7800FLV field-emission scanning electron microscope (JEOL, Peabody, MA, USA) at a voltage of 20 kV and a working distance of 10 mm.

### 5.8. Statistical Analyses

Quantitative data are expressed as mean ± standard deviation (SD). Statistical analyses between experimental groups were performed with Student’s *t*-test for independent samples or one-way analysis of variance (ANOVA) followed by post-hoc pairwise comparisons by Tukey test with GraphPad Prism version 10.0.3 for Windows, (GraphPad Software, Boston, MA, USA), *p*-value ≤ 0.05 was set as the level for statistical significance.

## Data Availability

*Amelx-iCre*; *Adam10^fl/fl^* frozen mouse sperm is awaiting approval for distribution at Mutant Mouse Resource and Research Centers (MMRRC). Additional data used to support the findings of this study are available from the corresponding author upon request.

## Supplementary Materials

The following supporting information can be downloaded at: https://www.mdpi.com/article/10.3390/ijms252313184/s1.
