# Supplementary material for: ADAM10 Expression by Ameloblasts Is Essential for Proper Enamel Formation
Source: Int J Mol Sci. 2024 Dec 7;25(23):13184. doi: 10.3390/ijms252313184 (PMC11641948; doi:10.3390/ijms252313184)
Supplement: Supplementary file 1 [file ijms-25-13184-s001.zip › ijms-3337133-supplementary.pdf]

International Journal of *Molecular Sciences*

MDPI

Supplementary Materials

ADAM10 Expression by Ameloblasts is Essential For Proper Enamel Formation

Shifa Shahid[1], Yuanyuan. Hu[2], Fatma Mohamed[1], Lara Rizzotto[1], Michelle C. Layana[1], Daniel T. Fleming[1], Petros Papagerakis[3], Brian L. Foster[1], James P. Simmer[2], John D. Bartlett[1]*

[1]Division of Biosciences. Ohio State University, College of Dentistry, 305 W. 12th Ave. Columbus, OH 43210, USA; Shahid.30@osu.edu (S.S.); fmohamed@umich.edu (F.M.) rizzotto.1@osu.edu (L.R.); layana.2@osu.edu (M.C.L.); fleming.606@buckeyemail.osu.edu (D.T.F); foster.1004@osu.edu (B.L.F.); Bartlett.196@osu.edu (J.D.B.)

[2]Department of Biologic and Materials Sciences, University of Michigan School of Dentistry, 1011 North University, Ann Arbor, MI, 48190, USA; yyhu@umich.edu (Y.H.); jsimmer@umich.edu (J.P.S.)

Laboratory of Precision Oral Health and Chronobiology, Faculty of Dentistry, Laval University, Dental Medicine Pavilion, 2420, rue de la Terrasse, Quebec City, QC G1V 0A6, Canada; petros.papagerakis@fmd.ulaval.ca (P.P.)

*Correspondence: bartlett.196@osu.edu; Tel: 1-614-292-7585

Contents:

Figure S1. Assessment of mouse organs for *Cre* expression

Figure S2. Assessment of mouse tissue sections for *Cre* expression

Figure S3. Quantification of *Ambn, Enam Col17a1*, *Mmp20 and Relt* gene expression levels in *Adam10*$^{fl/fl}$ (control) and *Adam10* cKO (experimental) mice.

Table S1. TaqMan probes for qPCR analyses of gene expression

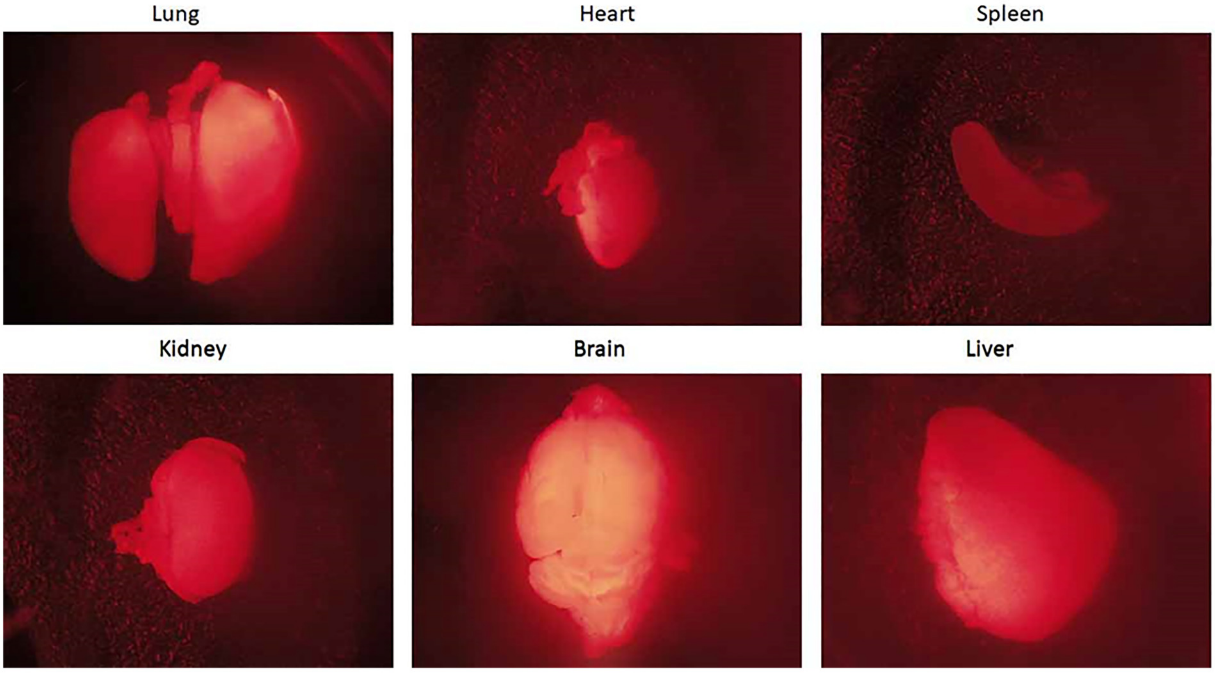

Figure S1. Assessment of mouse organs for *Cre* expression. *Amelx*-i*Cre* mice were crossed with the mouse mT/mG reporter strain so that *Cre* expression would cause tissues to stain with green fluorescent protein (GFP) whereas areas of red florescence indicate that no Cre recombination occurred. GFP staining was absent in the lung, heart, spleen, kidney, brain and liver.

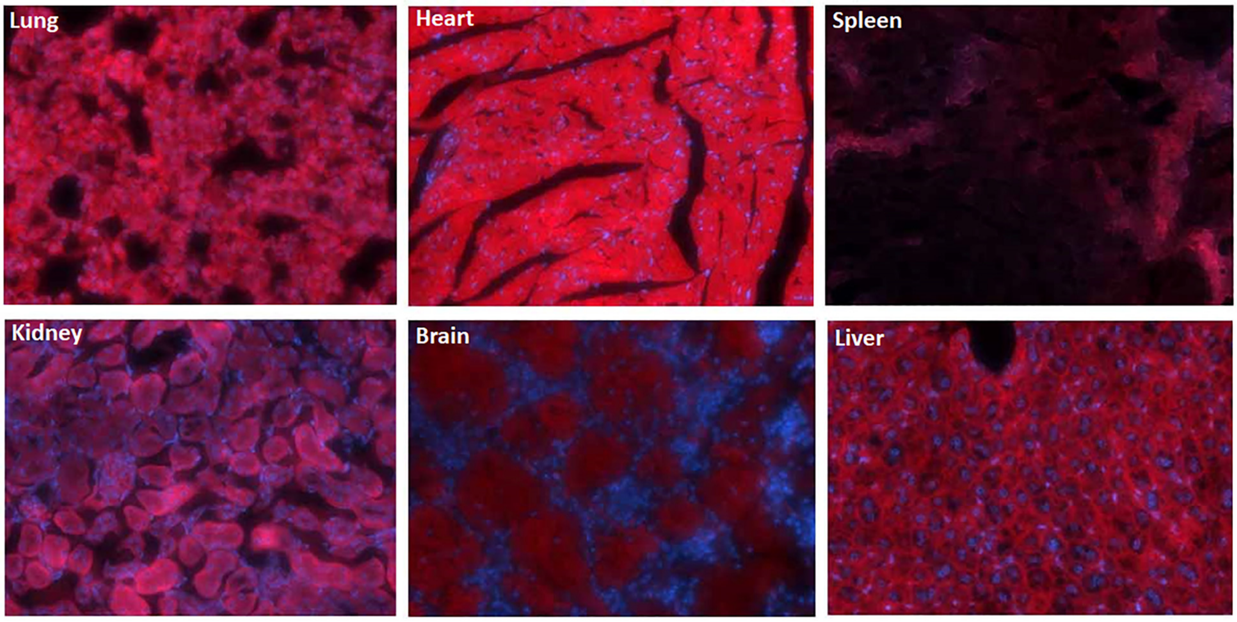

Figure S2. Assessment of mouse tissue sections for *Cre* expression. *Amelx*-i*Cre* mice were crossed with the mouse mT/mG reporter strain so that *Cre* expression would cause tissues to stain with green fluorescent protein (GFP) whereas areas of red florescence indicate that no *Cre* recombination occurred. GFP staining was absent in the lung, heart, spleen, kidney, brain and liver tissue sections.

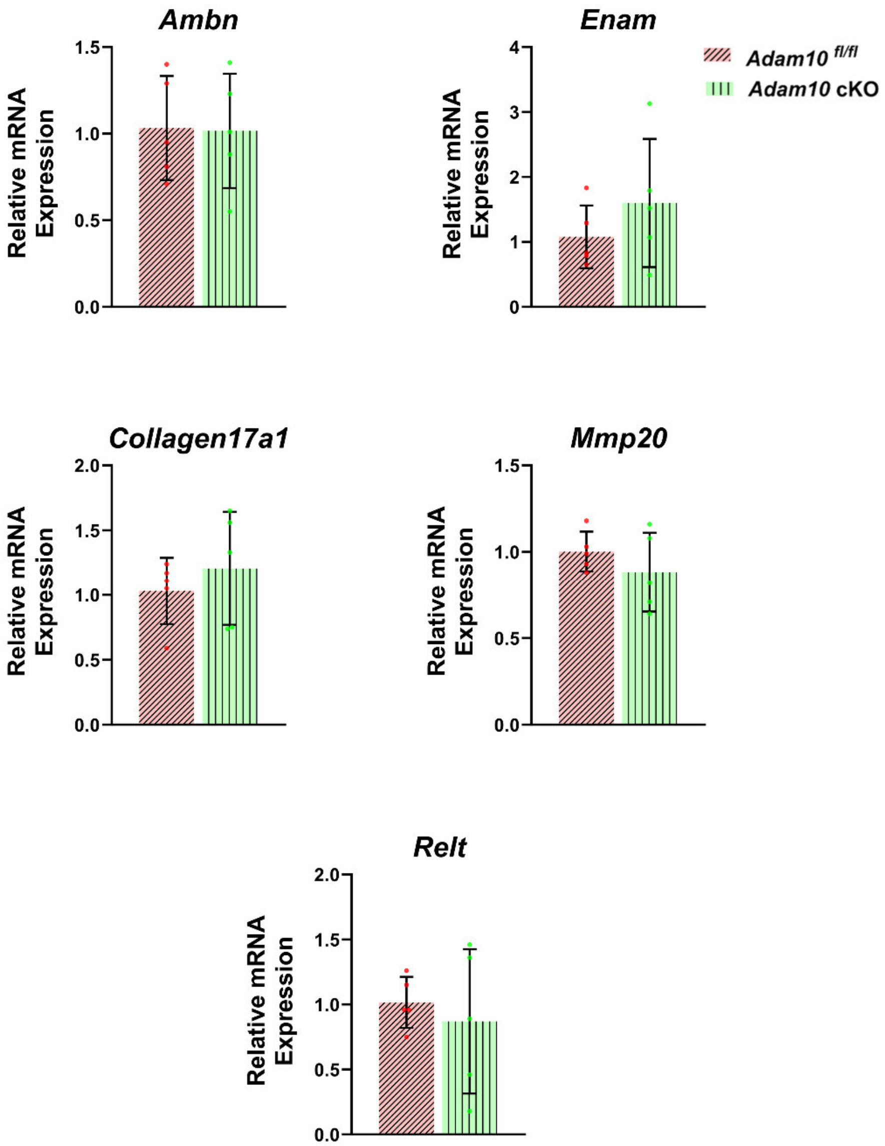

Figure S3. Quantification of *Ambn, Enam Col17a1*, *Mmp20 and Relt* gene expression levels in *Adam10$^{fl/fl}$* (control) and *Adam10* cKO (experimental) mice. Gene expression levels were assessed in day-5 first molar enamel organs by qPCR analyses to determine if ADAM10-mediated cell signaling effects the expression of genes that when mutated can cause enamel defects. Compared to the *Adam10$^{fl/fl}$* control, no significant difference in expression was observed in *Adam10* cKO molars among the genes encoding enamel matrix proteins (*Ambn*, *Enam*, *Mmp20*) or cell surface proteins (*Relt, Col17a1*). Therefore, ADAM10 signaling does not appear to significantly regulate the expression of these genes.

Table S1. TaqMan probes for qPCR analyses of gene expression

Gene	Assay ID	RefSeq	Probe Context Sequence
Glyceraldehyde-3-phosphate dehydrogenase (Gapdh)	Mm99999915_g1	NM_001289726.1	GGTGTGAACGGATTTGGCCGTATTG
A disintegrin and metallopeptidase domain 10 (Adam10)	Mm00545742_m1	NM_007399.3	TGGATTGTGGCTCACTGGTGGGCAG
Ameloblastin (Ambn)	Mm00477486_m1	AK017250.1*	TTTGAGCCTTGAGACAATGAGACAG
Amelogenin (Amelx)	Mm00711642_m1	NM_001081978.2	GCTATGCCCCTACCACCTCATCCTG
Collagen, type XVII, alpha 1 (Col17a1)	Mm00483525_m1	NM_001290825.1	ACCGAGAGAATTGTCACGGAAACAG
Enamelin (Enam)	Mm00516922_m1	NM_017468.3	GGCCAATTCCACAGAGGGGACCACC
Matrix metallopeptidase 20 (Mmp20)	Mm00600244_m1	NM_013903.2	TGAAACTGGAGATCATGGGGATTCC
RELT tumor necrosis factor receptor Relt	Mm00723872_m1	NM_177073.6	ACTGGTGGCTGTGATGAGTCAGGGC

* GenBank ID
